# Exploring Associations Between Heart Disease and Its Risk Factors and Alzheimer's Disease and Related Dementia In American Samoans

**DOI:** 10.1002/alz70860_107768

**Published:** 2025-12-23

**Authors:** Toaiva Hall, Vaatausili Tofaeono

**Affiliations:** ^1^ American Samoa Community Cancer Coalition, Nu'uuli, Pago Pago, American Samoa

## Abstract

**Background:**

There have been several studies that have provided evidence of the link between cognitive impairment and heart disease, while other research has provided results contradicting such an association. Regardless, significant disparities in both Alzheimer's disease and related dementias (ADRD) as well as cardiovascular disease (CVD) exist especially for Native Hawaiian and Other Pacific Islanders (NHPIs). The purpose of this study is to explore the association between CVD and ADRD risk specifically in Samoans, with an emphasis on understanding the effect of CVD risk factors on ADRD risk.

**Method:**

This study is a retrospective secondary data analysis that utilized data collected by the American Samoa Community Cancer Coalition's Puipui Malu Manatu research study (RF1AG075904) on ADRD, currently being conducted in the US territory of American Samoa. Chi‐square test of independence was used to determine the association between ADRD risk level and heart disease status. De‐identified data from 680 adults aged at least 50 years of Samoan ancestry was used. Preliminary ADRD risk level (Low, Intermediate, High) was determined via ranking the scores of the primary three objective cognitive impairment assessments (Cognivue, Montreal Cognitive Assessment, and the Number Symbol Coding Test) administered to study participants. These risk level scores will be validated using blood biomarker results from blood samples obtained from participants. Multiple linear regression will be used to understand the relationship between CVD risk factors (age, sex, physical activity, diet, alcohol and tobacco use, hypertension, high cholesterol, and family history of heart disease) and validated ADRD risk level using blood biomarker data collected by ASCCC. Data collection is ongoing.

**Result:**

Of 680 participants, only 57 reported medically diagnosed heart disease status. Initial results from Chi‐square analysis show no association between heart disease and ADRD risk level (*p* = 0.14). Data collection and analysis for results for blood biomarker data is still ongoing.

**Conclusion:**

There appears to be no association between heart disease and ADRD risk in Samoan adults in American Samoa. Validated ADRD risk levels using blood biomarker data is needed to explore possible association between effect of CVD risk factors on ADRD risk in this population. Data collection is ongoing.

